# Remote Monitoring in Adults With Congenital Heart Disease: A Patient Experience Study

**DOI:** 10.1177/23743735251399968

**Published:** 2026-01-30

**Authors:** Kuldeepa Veeratterapillay, Isobel Chaudhry, Debbie McParlin, Bill Chaudhry, Louise Coats

**Affiliations:** 1 Adult Congenital Heart Unit, 567753 Freeman Hospital, Newcastle upon Tyne Hospitals NHS Foundation Trust, Newcastle upon Tyne, UK; 2 Population Health Sciences Institute, Newcastle University, Newcastle Upon Tyne, UK; 3 204274 Institute of Global Health, University College London, London, UK; 4 Biosciences Institute, 105562 Newcastle University, Newcastle Upon Tyne, UK

**Keywords:** adult congenital heart disease, remote monitoring, wearable technology, patient experience, surveillance

## Abstract

Surveillance for long-term complications in adults with congenital heart disease (ACHD) presently relies on outpatient visits. Self-monitoring is increasingly being utilised to reduce the burden of care. This study explores the experiences of ACHD patients undertaking self-monitoring using wearable technology. A prospective observational cohort study was undertaken with ACHD patients across the United Kingdom. A CardiacSense watch recording vital signs and user-initiated electrocardiograms (ECGs) was sent to participants via post. Watch activity was monitored, and participants completed a postmonitoring questionnaire. Fifty-three individuals were screened. Thirty-three eligible participants were sent watches, and 24 (75% female, median age 44.5 years) completed the study. Participants wore watches for 12.3 days (range 1.6-29.8). Younger age was associated with shorter duration of watch-wearing (*R* = 0.413, *P* = .05), and men tended to record more ECGs than women (*P* = .009). Factors affecting engagement included comfort, digital literacy, and reported levels of anxiety and depression. ACHD engaged well with self-monitoring. Several factors, such as age, comorbidities, and digital literacy, influenced patient participation. Recommendations, grounded in patient experience, are suggested to ensure optimal adoption of self-monitoring in this population.

## Introduction

The key function of outpatient care for adults with congenital heart disease (ACHD) is surveillance for late-onset complications. However, present ambulatory care is not patient-centered, costly to providers, and associated with high levels of non-attendance and disproportionate interim visits to the emergency department and general practitioner.^1,2^ Significant healthcare disparities also exist between different demographic groups when accessing this model of care.^3^ Addressing these problems is important as the population of adults living with congenital heart disease (CHD) now exceeds the childhood population and continues to develop in complexity.^4^

Determining well-being in the clinic relies on clinician interviews alongside measurement of physiological parameters (blood pressure, heart rate, oxygen saturation, electrocardiogram [ECG], and echocardiography). However, detection of problems at clinic visits is opportunistic and can be confounded by healthcare anxiety.^5^ This may lead to delayed diagnosis or initiation of inappropriate therapy. The development and availability of wearables, biosensors, and smartphone-based applications over the last decade now offers the possibility for more comprehensive surveillance strategies, which, when combined with virtual appointments, have the potential to move ambulatory healthcare out of the hospital setting and into the patient's own environment.^6^ However, clinicians worry that increasing patient involvement in monitoring may exacerbate health anxiety for those living with ACHD,^7,8^ acknowledging the psychological and behavioral dimensions of self-monitoring, which may impact engagement alongside other aspects such as digital literacy.^9^

The aim of this study is to understand the ACHD patient's experience of supported self-monitoring for arrhythmia with wearable technology. Very few studies examine patient perspectives of remote rhythm monitoring with wearables, and none in the setting of ACHD, yet increasingly wearable technology is integrated into clinical use. This study tests the feasibility of this approach in ACHD and provides information that should inform future testing and roll-out of this technology.

## Methods

A prospective observational cohort study was undertaken with participants living across the United Kingdom.

### Study Population

Participants were recruited from a closed social media group administrated by a national patient organization for ACHD. Interested individuals were invited to contact the research team and then sent a screening questionnaire. Inclusion criteria included: age over 18 years, a known diagnosis of CHD, ability to understand written English, and access to a suitable mobile phone. Exclusion criteria included hospital monitoring in the preceding 12 months.

### Data Collection

A CardiacSense watch was sent to each eligible participant by post, and they were asked to wear it for 2 weeks. The participant interacted with the watch via a paired mobile phone app guided by a quick-start guide explaining the set-up. Watch activity was monitored by the research team via a web-based physician portal. Watches measured heart rate, respiratory rate, and oxygen saturation in a real-time fashion. These parameters were sent automatically to the portal. Participants could record a user-initiated ECG, which was then digitally uploaded to the portal. Metrics included time spent wearing the watch, number of patient-initiated recordings, and duration of apparent arrhythmic episodes. The research team was available for clinical concerns, and the CardiacSense team provided technical support. On completion of the monitoring period, participants were sent a questionnaire to complete.

### Questionnaires

Questionnaires were created in Qualtrics XM™ (London, UK) and provided to participants via email link and QR code. The final questionnaire covered demographic, clinical, and quality of life aspects, including patient-assessed New York Heart Association (NYHA) class, EuroQol-5 Dimensions-5 Levels (EQ-5DL),^10,11^ a series of numerical rating scale questions [0—most negative to 100—most positive], and watch experience questions using a Likert scale (Supplemental File 1). Likert scale question components were developed by the research group (medical professionals, a specialist nurse, researchers, and industry specialists) following an online focus group comprising adults with CHD recruited through the Sommerville Foundation that focused on patient experiences with remote monitoring. The questionnaire was reviewed by 2 ACHD patients prior to rolling out. A single open-ended question was included to enable participants to reflect in their own words on any other information they wished to feed back.

### Data Analysis

To explore the association between watch experience and demographic, clinical, and quality of life factors in this numerically small cohort, responses were grouped into a limited number of categories. European Society of Cardiology (ESC) Complexity and American Heart Association (AHA) physiological classes were assigned according to the information participants provided, with physiological classes grouped A/B and C/D.^12,13^ The 20 Likert scale questions were condensed into the following 4 general areas of interest, agreed by the research team, each composed of 5 questions with an average score taken in each group:

- Understanding of watch technology
- Confidence in the watch
- Understanding of health
- Watch comfort

Data from the open-ended question component of the questionnaire were analysed using thematic analysis.^14^ Data was managed in NVivo for Mac (version 14.24.0). Due to the relatively small data set, coding was undertaken by one author only [IC] and then codes and emerging themes were iteratively discussed among the research team alongside the quantitative information from the watch usage data and questionnaire responses to agree on final themes.

### Statistical Analysis

Analyses were performed using the R-package version 4.3.2 (R Foundation for Statistical Computing, Vienna, Austria). Normality was tested using Shapiro-Wilk's test. Variables were presented as mean ± standard deviation if normally distributed or median and range if non-normally distributed. Categorical variables were presented as numbers and percentages. Chi-square and Fisher's exact tests for small samples were used to compare categorical variables. Analysis of variance was used to compare normally distributed continuous variables, and the Mann-Whitney *U* test was utilized for nonnormally distributed variables. Pearson's and Spearman's rank correlations were used to test associations between normally distributed, nonnormally distributed, and ordinal variables, respectively. A 2-sided *P*-value of <.05 was considered indicative of statistical significance. Only 1/25 participants did not complete the postmonitoring questionnaire following the period of monitoring; this data was excluded list wise. All other questionnaire components were completed by all participants.

## Results

### Study Population

Of the 53 respondents who sent the screening questionnaire, 33 participants were sent watches, and 25 completed the monitoring period (Supplemental Figure 1).

**Figure 1.**
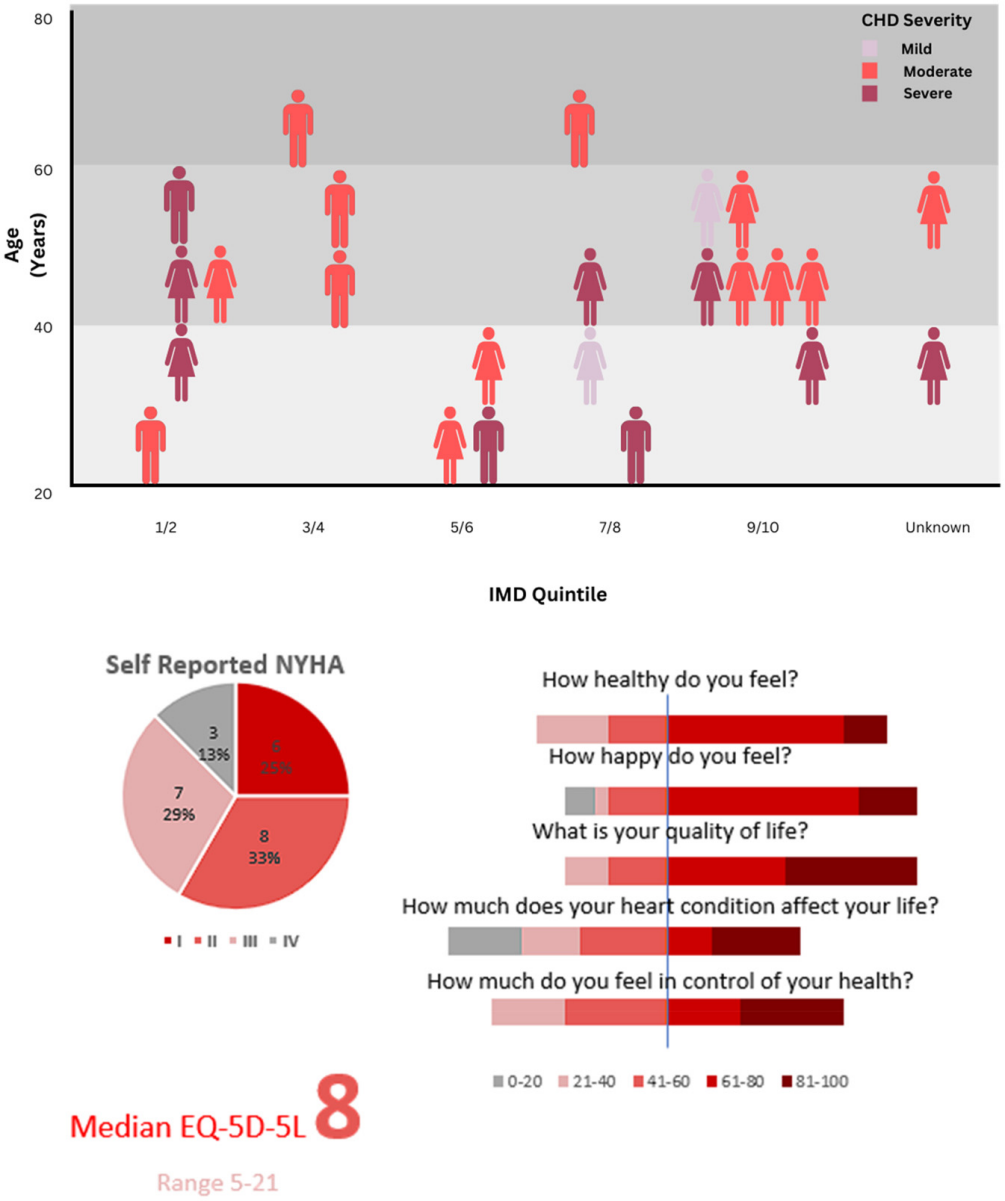
Demographics of watch wearing participants, their symptoms and health perceptions.

Abbreviations: CHD, congenital heart disease; IMD, Index of Multiple Deprivation (quintile 1/2 most deprived, quintile 9/10 least deprived); NYHA, New York Heart Association functional class; EQ-5D-5L, EuroQoL – 5 dimensions – 5 levels; NRS, Numerical Rating Score.

Twenty-four participants returned the post-monitoring questionnaire. They were predominantly female with moderate/severe complexity CHD, physiological class C/D (Figure 1). Median age was 44 years, and all but one respondent was of White British ethnicity. Median index of multiple deprivation (IMD) decile 6.5 [1-10]. From the available metrics, no differences were seen between those returning the questionnaire and those excluded or withdrawing from the study (Table 1).

**Table 1.** Comparison of Demographics Between Those Completing the Study and Those Withdrawing or Excluded.

	Excluded and withdrawals (*n* = 29)	Participants completing questionnaire (*n* = 24)	*P*
Female sex	20 (69%)	18 (75%)	.627
Age (years)	50 (17-71)	44 (20-65)	.215
CHD regions	10/12	9/12	
White British	24 (82.8%)	23 (95.8%)	.204

Abbreviation: CHD, congenital heart disease.

A total of 17/24 (71%) participants already self-monitored with a range of devices (KardiaMobile^®^ 4, Apple Watch^®^ 5, Fitbit^®^ 5, Garmin^®^ 1, Withings^®^ ScanWatch 1, Pacemaker recording system 2, home blood pressure device 7, and pulse oximeter 6). A total of 8/24 (33%) participants used multiple devices.

### Participant Watch Use

Participants wore their watches for a mean of 12.3 days (range 1.6-29.8), with continuous monitoring available for 294 h (39-714), 92% (61-100) of the available time. A total of 14/24 participants undertook patient-activated ECG recordings on 5 occasions (range 0-30). Seven participants reported symptoms (4 chest pain, 2 palpitations, and 1 dizziness), but only 6 performed ECGs; the user interface did not enable direct symptom rhythm correlation. In 2, who reported palpitations, possible atrial fibrillation, and bigeminy were seen, respectively. Younger patients tended to wear the watch for fewer days (*P* = .06, *r* = .398, 95% CI, 0.073-0.694) and fewer hours overall (*P* = .05, *r* = .413, 95% CI, 0.104-0.677). IMD was not associated with watch-wearing. Participant withdrawals occurred across the timeline of the study.

### Emerging Themes in Experiences With Remote Monitoring

Six themes concerning supported self-monitoring with the CardiacSense watch emerged following iterative discussion of quantitative data analysis alongside the qualitative data coding: administrative burden, comfort, convenience, confidence in digital literacy, and anxiety. The themes are discussed below, with representative quotes presented in Table 3.

- Administrative burden. Four participants failed to complete the screening questionnaire, and one, despite undertaking monitoring, failed to complete the final questionnaire. Four patients withdrew or were withdrawn due to a lack of clarity regarding the compatibility of the watch with implanted medical devices. Participant feedback indicated difficulty processing some of the information provided (Table 3).
- Comfort. One participant withdrew after receiving the watch due to it being too large for their wrist. A range of experiences relating to the size, material, and aesthetic nature of the watch were reported. Importantly co-existence of other long-term conditions, a frequent feature in ACHD, impacted on watch comfort (Table 3). A tendency towards less time wearing the watch was observed in those who reported more pain (as a dimension of EQ-5D-5L, *P* = .054, *r* −.407, 95% CI, −0.733 to −0.014). Participants who reported a strong sense that their heart affected their life were more likely to experience discomfort (*P* = .043, *r* = –.417, 95% CI, −0.724 to −0.029).
- Convenience. One participant withdrew after receiving the watch due to illness and feeling unable to contribute. A further participant reported not wearing the watch when distracted by competing activities, while another removed the watch when their child experienced gastroenteritis. Generally, participants wore their watches most of the time, as indicated by the quantitative data, but at times of stress or when there were competing activities, information may have been lost. Consistent with this, it was observed that those with advanced physiological class demonstrated a wider range of watch-wearing behavior than those with no symptoms (Table 2).

**Table 2.** Variation in Watch Wearing Behavior with AHA Physiological Class.

AHA physiological class	A/B (*n* = 4)^a^	C/D (*n* = 19)^b^	*P*
Days watch worn Median (range)	13.9 (13.3-14.6)	9.0 (1.6-29.8)	0.123
Hours watch worn Median (range)	372 (343-463)	252 (39-720)	0.168

Abbreviations: AHA, American Heart Association; ICD, implantable cardiac defibrillator.

^a^ Operation in previous year (*n* = 1), ^b^Operation in previous year (*n* = 4), arrhythmia (*n* = 17), heart failure symptoms (*n* = 8), pacemaker or ICD (*n* = 7), hypertension (*n* = 5).

**Table 3.** Participant Quotes Identifying Key Themes Relating to Engagement and Experience With Remote Monitoring With the CardiacSense Watch.

Administrative burden
“*I didn’t realise that I could record data without the app open*” “*The watch itself wasn't too difficult to manage, however the software was*”
Comfort and useability
“*Having rheumatoid arthritis, the strap was hurting my wrists some of the time*” “*I did find the face quite large which looked comical due to my small wrists but it was accurate*” “*I suffer from eczema and found that I developed a rash on my wrist under the watch which made me less inclined to wear it*” “*As a dyslexic/dyscalculic would have been easier with a digital face as unable to read the clock properly*”
Convenience
“*I was very happy that I could do an ECG whenever I wanted, and the heart rate was very accurate*” “*I found having to charge it for an hour a day very inconvenient, not least because I'd forget to put it back on*” “*The readings seemed to take a long time to complete*” “*(it was) difficult to take a reading while walking/doing an activity*”
Confidence
“*I don’t believe the days reading as often they were quite low and I don’t believe this to be accurate but I maybe wrong as nothing to compare to*” “*The readings were often significantly different to my other wearables …* *I didn't know which reading to trust!*” “*The option to get a monthly report and send data to your doctor is great*” “*If the measurements could be proven to be much more accurate than the commercial alternatives, then I might be persuaded to switch*”
Digital literacy
“*It took me several hours to set the software up, and this was with considerable techy help from the family*” “*The watch initially failed after a software update, and I waited over the weekend for advice so couldn't use it for 2 days*” “*Initially I found that my phone wasn't adequate for the software, so I had to borrow a phone*”
Anxiety
“*Some of the ECG lines looked quite alarming, both on screen and on the reports*” “*Had I been inclined to be anxious about my oxygen levels I would have been very worried by this*”

Participants enjoyed using the watch but also expressed impatience and questioned its applicability (Table 3).

**Figure 2.**
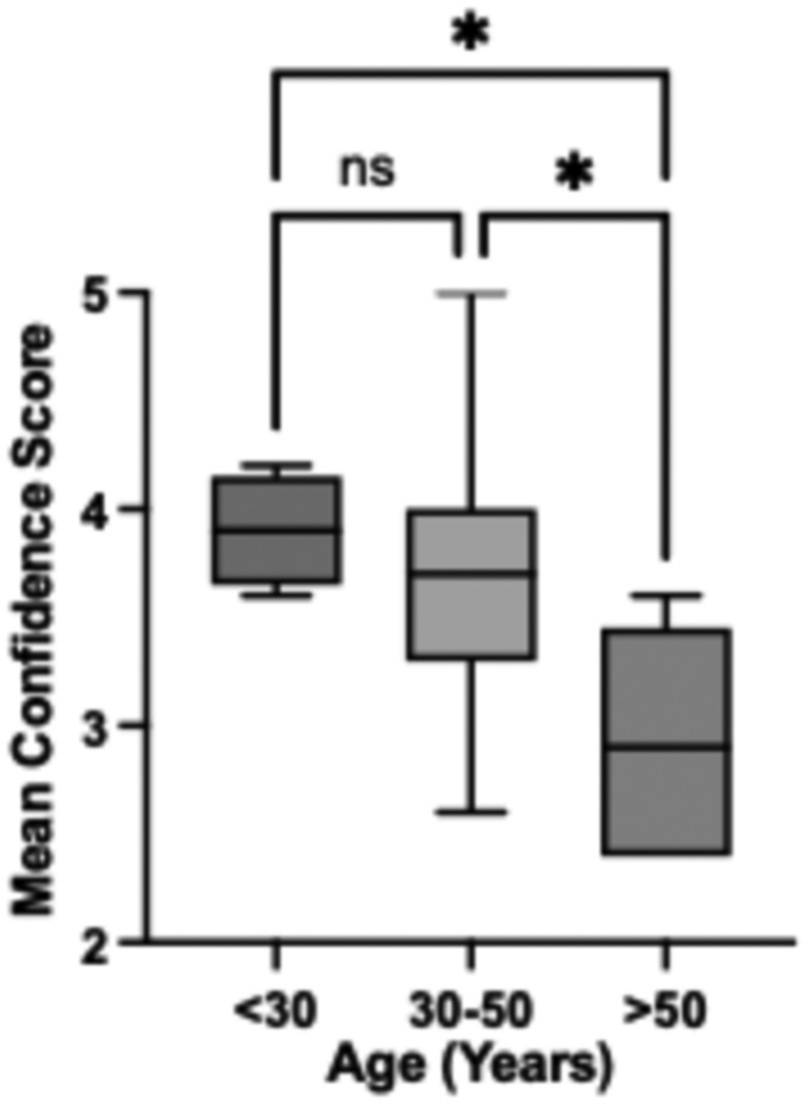
Confidence in watch score according to age category [Kruskal Wallis *p*=0.0152 with posthoc Dunn's test, ns: not significant, * *P* < .05].

- Confidence. Many participants invested time in understanding how to use the watch and the nature of its monitoring purpose, but expectations of their own measurements and comparison with existing wearables, often with different functionality, were frequent and affected confidence; some features of the watch reassured (Table 3). Older participants expressed less confidence in the watch (*P* = .007, *r* = –.537, 95% CI, −0.813 to −0.132) and tended to be less understanding of the technology (*P* = .064, *r* = –.384, 95% CI, −0.743 to 0.132). Lack of confidence was significant in those over 50 years (Figure 2).
- Digital literacy. One participant withdrew after receiving the watch due to technical difficulty with set-up. A requirement for a software update for some participants meant 7 participants sought technical support and then continued monitoring. One watch required replacement due to a critical error. Participants described a range of issues, including the value of both informal and formal support, the effects of delays, and compatibility with existing hardware (Table 3).
- Anxiety. One participant withdrew after receiving the watch due to high preexisting health anxiety, with the set-up causing additional stress. A further reported that their health anxiety was high following surgery, but appreciated the technical support provided and completed the monitoring period. While no participants reported exacerbation of anxiety with watch wearing, some suggested elements of the monitoring could have provoked concern (Table 3). Participants who reported high levels of anxiety/depression (as a dimension of EQ-5D-5L) in fact tended to be more confident with the watch (*P* = .018, *r* = .480, 95% CI, 0.119-0.730), although there was no suggestion that this related to feeling in better control of their health.

## Discussion

The present feasibility study shows that in a group of ACHD, supported self-monitoring with a watch can facilitate reporting of vital signs and provide the ability for the patient to act on symptoms and initiate more detailed physiological recordings that can be delivered to the clinician. Despite instructions, however, individuals had different degrees of health and digital literacy and used remote monitoring in ways that were convenient to them, irrespective of their cardiovascular risk. Participants were from a range of ages, socio-economic backgrounds, and disease complexities; however, most were white and female, indicating a need for further studies among those of different ethnicities.

Arrhythmia is frequent among ACHD and may be due to congenitally abnormal conduction pathways or inherent structural or hemodynamic issues. Risks may further be exacerbated by surgical scarring, medication, electrolyte disturbances, or systemic illness.^15^ Consequences range from impaired quality of life, acceleration of heart failure, hemodynamic instability, and sudden cardiac death. It is estimated that 50% of 20-year-olds with CHD will develop an atrial tachyarrhythmia during their lifetime.^16^ In some cohorts, high prevalence of arrhythmia has led to new continuous monitoring surveillance approaches that have demonstrated a change in clinical management through early detection.^17,18^ These approaches almost certainly provide greater diagnostic yield than routine annual Holter monitoring and thus hypothetically may reduce sudden cardiac death risk.^19^ However, randomized controlled trials have never been performed, and therefore, key cardiovascular and all-cause endpoints have not been evaluated. In the present study, it was notable that those with pain, more advanced symptoms, or co-existent long-term conditions, who may be a group at greater clinical risk, were more likely to exhibit reduced or inconsistent patterns of monitoring. This may indicate a need for tailored support in these groups or indeed in others who may experience temporary deterioration in symptoms. In addition, illness identity, which has been shown to influence health care utilization in ACHD, may have impacted watch-wearing, as participants who reported a strong sense that their heart affected their life were more likely to experience discomfort.^20^ This may be because they are more susceptible to heightened sensory awareness or perhaps perceive a loss of autonomy with monitoring that increases discomfort.

Over a third of ACHD patients experience clinical levels of anxiety or depression or both; this is associated with reduced quality of life and perceived health status.^21^ These patients have a higher frequency of health service usage.^22^ Clinicians perceive remote monitoring may exacerbate anxiety among patients, and in the context of atrial fibrillation, has been associated with higher rates of symptom monitoring and preoccupation, treatment concerns, and rhythm-related healthcare use.^7,8^ Interestingly, we found that those with higher levels of anxiety and depression felt more confident using the watch. Correlation of higher anxiety with greater confidence in watch use seems counterintuitive. However, health anxiety is associated with increased healthcare utilization across multiple settings.^23^ Additionally, health anxiety can arise among those who access health information through online searching (cyberchondria) and necessarily have high “digital confidence.”^24^ The health belief model supports this finding as it would suggest that anxious individuals perceive themselves to be at more risk and will lean towards an external locus of control, in this case, the watch, where the feedback and reassurance it provides manages their uncertainty and boosts self-efficacy.^25,26^ So, they actually feel more confident using it compared to less anxious individuals.

E-Health smart technology used in the optimization and monitoring of heart failure for those with systemic right ventricles was found to make patients feel more secure, although a small minority still feel nervous.^27^ However, again, prospective randomized controlled studies are essential to determine whether these observations are simply a product of selection bias and determine and whether behavior is sustained over the longer term.

Overall, with the provision of technical support, adherence rates to watch monitoring in our study were high. This is consistent with other prospective studies using eHealth and telemonitoring in ACHD,^17,28^ and is explained by the high digital literacy of a relatively young adult population.^29^ The vast majority own a smartphone, and while only a few presently engage in mobile Health, most are willing to start.^30,31^ In the present study, only older participants tended to be less confident about the watch. Existing monitoring devices, such as Holter or mobile telemetry devices, and event monitors, have suboptimal compliance because of their difficulty in use, interference with the patient's daily activities, and skin irritation leading to premature device removal.^32^

Novel wearables, which require less interaction and include smartphones, watches, and belts based on ECG and photoplethysmography, have good levels of acceptability to patients, as we have generally seen.^33^ One issue, however, is signal quality, which can be affected by motion artefacts. In ACHD, wearables must reliably detect rhythms and enable definite symptom rhythm correlation, which underpins treatment decisions in this population.^34,35^

Event recording functionality, individualized thresholds for rhythm detection, and home telemonitoring have already been integrated into modern pacemakers and defibrillators and demonstrate benefit in the ACHD population.^36,37^ Interestingly, among those with pacemakers, the vast majority prefer home monitoring to face-to-face review and are confident the technology is safe. A third, however, would like more reassurance that the technology is working all the time.^38^ Defining the groups that could gain clinical benefit, optimizing the technology for the ACHD group is critical. However, patient experience is the first step to ensure broad adoption and ensure new approaches do not widen health inequalities. In the general population, lower engagement with self-management interventions has been seen in the socioeconomically deprived and some ethnic minority groups.^39,40^ While this study in those living with ACHD did not identify socio-economic disparities, younger patients, older patients, and those with more advanced symptoms and other long-term conditions may need special consideration. Simplicity, comfort, convenience, and clinical and technical support are demonstrably essential.

Novel wearables such as watch-based rhythm monitoring could potentially provide a practical and cost-effective solution for detecting significant arrhythmias in ACHD. Clinical trials conducted to determine the populations and indications who could most benefit from such approaches must consider patient experience and engagement with these technologies to ensure solutions are accessible to all groups who have a clinical indication. The effects of body habitus, underlying bundle branch block, poor skin contact, and incorrect placement of mobile health devices are not explored here and also need further study in order to optimize use in ACHD. In addition, there is increasing evidence that wearable devices work differently in people with darker skin tones, due in part to technological limitations of photoplethysmography green light signalling.^41,42^ The Department of Health and Social Care in the United Kingdom recently published an independent report calling for concerted action by stakeholders to address this important issue, which has been shown to lead to diagnostic health inequalities.^43^

### Limitations

Research involving adult patients with CHD is inherently challenging due to the limited number of patients and the heterogeneity of their conditions and symptoms. This study represents a small sample and short follow-up period and is not powered to detect an outcome. The short duration of monitoring (median 12.3 days) may not reflect long-term engagement or sustainability. There is also potential for response bias and or social desirability bias, given a high proportion of participants were already engaged in self-monitoring, and participants were recruited via a patient organization. However, it nevertheless demonstrates the feasibility of the approach in a range of participants, highlighting individual needs that should be considered in clinical trial design and subsequent clinical roll-out. The study population represented a clinical group at high risk of cardiac arrhythmia and potentially heart failure; selection bias resulting from the recruitment strategy seems likely, as there was a disproportionate number of females aged 40 to 60 years from higher socioeconomic groups. Additionally, there was a high prevalence of existing self-monitoring. In ACHD, nonattendance at clinic and loss to follow-up are frequent and associated with lower socio-economic class.^2^ Further work needs to be undertaken, incorporating these groups to understand whether supported self-monitoring can improve engagement and access to healthcare, or whether, in fact, it may widen inequality. Future studies with a longer observation period would be valuable to determine whether patients adapt to home monitoring over time and to explore the potential for patient fatigue (a phenomenon where device usage declines over time).

#### Limitations of the Survey Tool

Although components of the survey were validated (EQ-5D-5L, NHYA), other questions were added for the purpose of this study. Grouping of Likert-scale items, on reflection, incorporated a series of constructs. For example, *Comfort* incorporated ideas about both the physical comfort of the watch, the duration of watch wearing, and interference in daily life. Posthoc assessment of Cronbach's alpha statistic reflected this and was measured at 0.487 for the comfort questions, 0.446 for the understanding technology questions, 0.571 for the understanding of health questions, and 0.481 for the confidence questions. It should be noted, however, that due to the small sample size, the reliability of Cronbach's alpha may be reduced due to random sampling error and specifically may underestimate the true population reliability.

No relationship was found between self-reported NYHA, EQ-5D 5L, linear analogue scale measures, or the self-reported domains (confidence, comfort, understanding of technology and health) and duration of watch wearing (days or minutes) or number of ECGS recorded. For self-reported domains, this may be a methodological issue and is consistent with limited construct validity reflected in the Cronbach alpha statistics; however, it may also reflect the fact that other factors not captured by this questionnaire influence watch wearing, which requires further study.

Informal pilot testing of these components was undertaken with a couple of ACHD patients, but robust prospective methodology, including aspects such as cognitive interviewing, item refinement, and reliability and validity testing, was not possible due to funding and time constraints and should be considered in future work.

## Conclusion

This is the first study to report patient experience and engagement with wearable technology for arrhythmia surveillance in ACHD patients. Age, symptoms, comorbidities, confidence with technology, and digital literacy influence patient participation with remote monitoring. In contrast to clinician perception, those with higher levels of anxiety tend to be more confident with remote monitoring.

Remote monitoring solutions must work out of the box, have a flexible design, be fast to charge, and be accompanied by in-person technical support to maximize adoption. Alternative approaches should be considered in subgroups who may be less able to engage.

These initial findings can be used to inform future clinical trial design or implementation strategies, but further work is needed to ensure the findings are generalizable to more diverse populations, as well as assessing issues that may arise during long-term follow-up before changing models of care for surveillance among ACHD patients.

## Supplemental Material

sj-docx-1-jpx-10.1177_23743735251399968 - Supplemental material for Remote Monitoring in Adults With Congenital Heart Disease: A Patient Experience Study Supplemental material, sj-docx-1-jpx-10.1177_23743735251399968 for Remote Monitoring in Adults With Congenital Heart Disease: A Patient Experience Study by Kuldeepa Veeratterapillay, MBBS, Isobel Chaudhry, BSc, Debbie McParlin, BSC Hons, Bill Chaudhry, MBBS, PhD, and Louise Coats, MBBS, PhD in Journal of Patient Experience
