# Supplementary material for: Remote Monitoring in Adults With Congenital Heart Disease: A Patient Experience Study
Source: J Patient Exp. 2026 Jan 30;13:23743735251399968. doi: 10.1177/23743735251399968 (PMC12861371; doi:10.1177/23743735251399968)
Supplement: sj-docx-1-jpx-10.1177_23743735251399968 - Supplemental material for Remote Monitoring in Adults With Congenital Heart Disease: A Patient Experience Study [file sj-docx-1-jpx-10.1177_23743735251399968.docx]

**Supplemental Material**

##
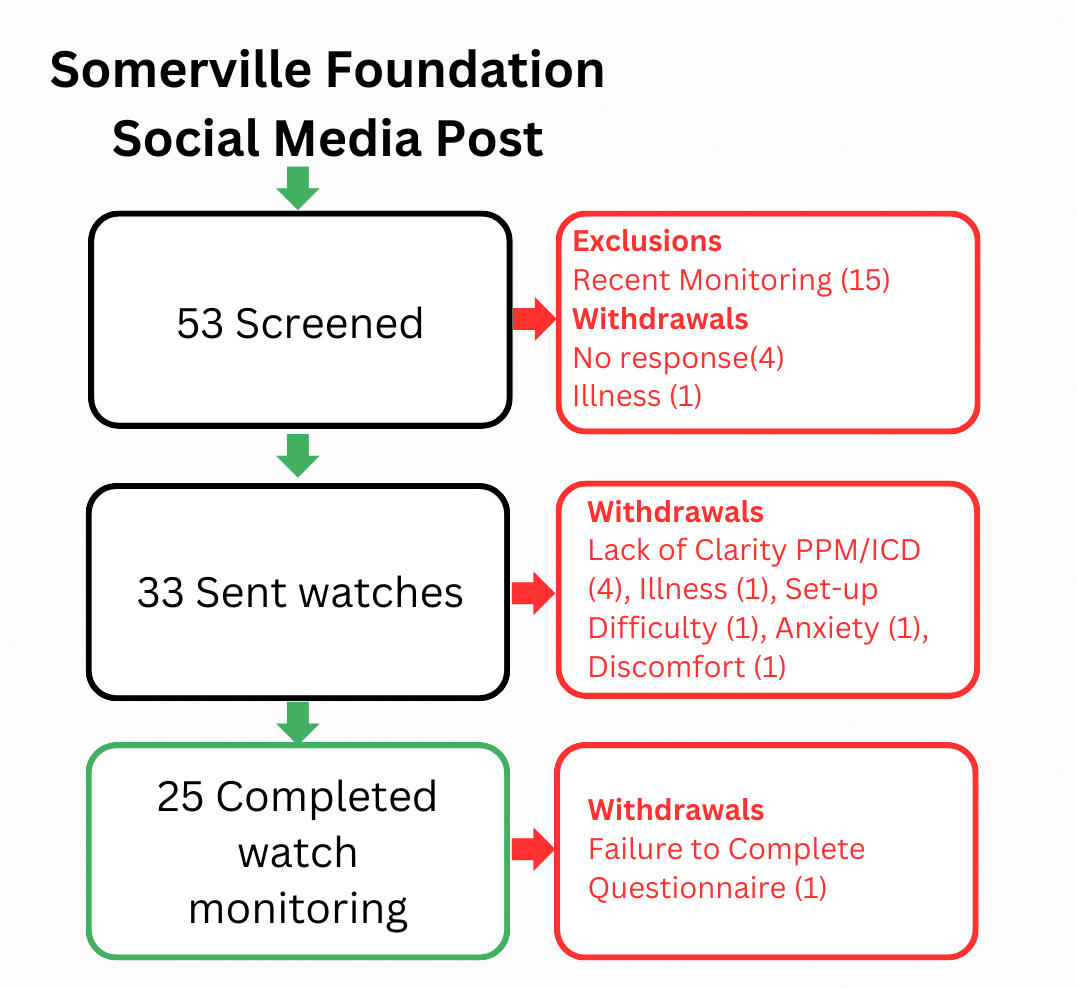


**Supplemental material Figure 1** Participant recruitment for watch monitoring [PPM: permanent pacemaker, ICD: implantable cardiac defibrillator]

## Online survey

Consent Statements Please initial each box below if you are in agreement with the statement

I confirm that I have read the information sheet dated 02/05/2023 (version 1.1) for the above study. I have had the opportunity to consider the information, ask questions and have had these answered satisfactorily.

I understand that my participation is voluntary and that I am free to withdraw at any time without giving any reason, without my medical care or legal rights being affected.

I understand that relevant sections of my data collected during the study may be looked at by regulatory authorities or individuals from the University, where it is relevant to my taking part in this research. I give permission for these individuals to have access to my data.

I understand that data collected by the Cardiacsense watch may be accessed by the company in line with the terms laid out in their Service Privacy Policy (<https://www.cardiacsense.com/services-privacy-policy/>)

I understand that the information collected about me will be used to support other research in the future and may be shared anonymously with other researchers.

I understand that if I withdraw from the study, any data collected up to the point of withdrawal may still be kept and used.

I understand that the information held and maintained by the University may be used to help contact me or provide information about my health status.

I agree to take part in the above study.

Consent Confirmation Please sign to confirm that you consent for this study

Consent Confimation Please type your name below

**GENERAL BACKGROUND**

Q1 Please enter today's date:

Q2 How old are you?

Q3 What is your sex?

- Male
- Female
- Non-binary / third gender
- Prefer not to say

Q4 What best describes your ethnic origin?

- White
- Black/African/Caribbean
- Asian (Indian, Pakistani, Bangladeshi, Chinese, any other Asian background)
- Mixed two or more ethnic groups
- Other (Arab or any others)
- Prefer not to say

Q5 Which statement best describes your current work situation?

- Employed (Full-time)
- Employed (Part-time)
- Unemployed or job-seeking
- Caregiver (eg. children, elderly)
- Homemaker
- Disability/government financial assistance
- Retired
- Student (Full-time)
- Student (Part-time)
- Other

Q6 What is the name of your congenital heart condition? If complicated describe as best you can.

Q7 Please describe the main treatments you have had through your life, including any procedures and surgeries (you can write up to 500 words)

Q8 Please tick any of the following that apply to you:

- I am waiting for an operation on my heart
- I have had an operation on my heart in the last year
- I suffer from problems with my heart rhythm
- I have a pacemaker or a defibrillator
- I have been told I have heart failure
- I take water tablets (diuretics eg. furosemide or bumetanide or similar)
- I have pulmonary hypertension
- My oxygen saturations are normally
- I have high blood pressure
- I am diabetic

**SELF-REPORTED NYHA**

Q9 Below you find four descriptions that describe different degrees in which you can be limited in your daily physical functioning. Which description is most applicable to you? Please only consider the limitations that you believe are caused by your congenital cardiac defect.

- I am not limited during physical activities. Ordinary physical activities do not cause extraordinary fatigue, palpitations or shortness of breath.
- I am slightly limited during physical activities. I do not experience any symptoms at rest, but ordinary physical activities cause extraordinary fatigue, palpitations or shortness of breath.
- I am considerably limited during physical activities. I do not experience any symptoms at rest, but less than ordinary physical activities cause extraordinary fatigue, palpitations or shortness of breath.
- I am very limited during physical activities and I am unable to be physically active without experiencing discomfort. Also at rest I experience fatigue, palpitations or shortness of breath and the discomfort increases when I am physically active.

**EQ-5D-5L**

Q10 Please select the ONE box that best describes your health TODAY.

 **MOBILITY**

- I have no problems in walking about
- I have slight problems in walking about
- I have moderate problems in walking about
- I have severe problems in walking about
- I am unable to walk about

Q11 Please select the ONE box that best describes your health TODAY.

 **SELF-CARE**

- I have no problems washing or dressing myself
- I have slight problems washing or dressing myself
- I have moderate problems washing or dressing myself
- I have severe problems washing or dressing myself
- I am unable to wash or dress myself

Q12 Please select the ONE box that best describes your health TODAY.

 **USUAL ACTIVITIES** (e.g. work, study, housework, family or leisure activities)

- I have no problems doing my usual activities
- I have slight problems doing my usual activities
- I have moderate problems doing my usual activities
- I have severe problems doing my usual activities
- I am unable to do my usual activities

Q13 Please select the ONE box that best describes your health TODAY.

 **PAIN / DISCOMFORT**

- I have no pain or discomfort
- I have slight pain or discomfort
- I have moderate pain or discomfort
- I have severe pain or discomfort
- I have extreme pain or discomfort

Q14 Please select the ONE box that best describes your health TODAY.

 **ANXIETY / DEPRESSION**

- I am not anxious or depressed
- I am slightly anxious or depressed
- I am moderately anxious or depressed
- I am severely anxious or depressed
- I am extremely anxious or depressed

NRS/SLIDER SCALE

Q15 **How healthy do you feel overall?**

100 means the best health you can imagine
 0 means the worst health you can imagine

Q16 **How happy do you feel overall?**

100 means the happiest you can imagine
 0 means the unhappiest you can imagine

Q17 **What is your overall quality of life?**

100 means the best quality of life you can imagine
 0 means the worst quality of life you can imagine

Q18 **How much does your heart condition affect your life?**

100 means your heart condition affects everything you do
 0 means your heart condition does not affect anything you do

Q19 **How much do you feel in control of your health?**

100 means you feel completely in control of your health
 0 means you feel you have no control over your health

 Likert Type Questions

Q20 You have recently used the Cardiacsense watch for two weeks. We would like to ask you some questions about your experience with it. Please answer the questions below by placing a cross in the box you most agree with.

The watch was comfortable to wear. [COMFORT]

I was not aware of the watch when I wore it [COMFORT]

I wore the watch all of the time [COMFORT]

Sometimes I worried whether I had used the watch correctly [UNDERSTANDING TECH]

I found the watch easy to use [UNDERSTANDING TECH]

I have been able to do my usual daily activities with the watch on [COMFORT]

The time I spent making recordings is acceptable [COMFORT]

Seeing my heart rate was reassuring [UNDERSTANDING HEALTH]

I felt more anxious about my health wearing the watch [UNDERSTANDING HEALTH]

When I wasn't sure about my watch, I knew who to contact [UNDERSTANDING TECH]

I understood the information the watch was telling me [UNDERSTANDING HEALTH]

I felt better about my health wearing the watch [UNDERSTANDING HEALTH]

I have had to go to hospital more often because of the watch [UNDERSTANDING IN TECH]

I was confident the watch would detect any problems with my heart [CONFIDENCE IN TECH]

I trust the doctors and nurses that are treating me [CONFIDENCE IN TECH]

Sometimes I’m not sure if the doctors got the recordings [CONFIDENCE IN TECH]

Seeing my oxygen levels was reassuring [UNDERSTANDING HEALTH]

The information that came with the watch was helpful [UNDERSTANDING TECH]

When I had technical problems, the doctors and nurses could help me [CONFIDENCE IN TECH]

I would recommend this watch to other patients [CONFIDENCE IN TECH]

[No, not at all, Slightly, Neutral, Somewhat, Yes, extremely]

Q21 Please write down anything else about having the Cardiacsense watch that you think is important for us to know

Q22 Finally, do you use any personal monitoring devices not specifically prescribed by the hospital, please list these here:
